# Profiling quality of care for patients with chronic headache in three different German hospitals – a case study

**DOI:** 10.1186/1472-6963-8-13

**Published:** 2008-01-16

**Authors:** Dieter Melchart, Anne Wessel, Ronald Brand, Stefan Hager, Wolfgang Weidenhammer

**Affiliations:** 1 Centre for Complementary Medicine Research, Department of Internal Medicine II, Technical University, Munich, Kaiserstr. 9, 80801 München, Germany; 2 Division of Complementary Medicine, Department of Internal Medicine, University Hospital Zurich, Switzerland; 3 formerly Dr. Köhler-Parkkliniken, Prof.-Paul-Köhler-Strasse 3, 08645 Bad Elster, Germany; 4 Migraine Hospital Dr. Brand, Ölmühlweg 31, 61462 Königstein, Germany; 5 Hospital for Traditional Chinese Medicine, Ludwigstrasse 2, 93444 Kötzting, Germany

## Abstract

**Background:**

Legal requirements for quality assurance in German rehabilitation hospitals include comparisons of providers. Objective is to describe and to compare outcome quality of care offered by three hospitals providing in-patient rehabilitative treatment exemplified for patients with chronic headache.

**Methods:**

We performed a prospective three center observational study on patients suffering from chronic headache. Patients underwent interventions commonly used according to internal guidelines of the hospitals. Measurements were taken at three points in time (at admission, at discharge and 6 months after discharge). Indicators of outcome quality included pain intensity and frequency of pain, functional ability, depression, quality of life and health related behavior. Analyses of differences amongst the hospitals were adjusted by covariates due to case-mix situation.

**Results:**

306 patients from 3 hospitals were included in statistical analysis. Amongst the hospitals, patients differed significantly in age, education, diagnostic subgroups, beliefs, and with respect to some pain-related baseline values (covariates). Patients in all three hospitals benefited from intervention to a clinically relevant degree. At discharge from hospital, outcome quality differed significantly after adjustment according to case-mix only in terms of patients' global assessment of treatment results. Six months after discharge, the only detectable significant differences were for secondary outcomes like improved coping with stress or increased use of self-help. The profiles for satisfaction with the hospital stay showed clear differences amongst patients.

**Conclusion:**

The results of this case study do not suggest a definite overall ranking of the three hospitals that were compared, but outcome profiles offer a multilayer platform of reliable information which might facilitate decision making.

## Background

In recent years there has been an increasing number of publications on profiling medical care providers with respect to quality of care, whether on the level of hospitals [1,2] or outpatient care [3,4], and predominantly dealing with methodological problems [5-7]. In Germany, Social Security Code requires hospitals to enable comparative analyses of their quality (Section 20, paragraph 1, Social Security Code IX) to assure quality of care and to demonstrate improvement in it. Since 2004, a comprehensive quality assurance program for hospitals for rehabilitation medicine has become accepted as a standard and is supported by all German statutory sickness insurances [8]. This program comprises aspects of quality concerning structure, process and outcome on the basis of conventional specialties in rehabilitative medicine (like orthopedic, cardiologic or psychosomatic rehabilitation, with others still under way). However, a minor number of hospitals are determined by their therapeutic concept (like Complementary and Alternative Medicine: CAM) and thus are confronted with many different diseases or, limited by a very special indication, like rehabilitation of chronic headache (usually assigned to "Neurological Rehabilitation"). Problems arise if such hospitals try to adopt the standard quality assurance program. Either the hospitals are not able to fulfill the requirements for structural quality concerning several rehabilitative disciplines or the less specific indicators of outcome quality do not fit the special conditions of the disease.

As a consequence, we performed a multi-center study on in-patients with chronic headache in three different hospitals providing unconventional rehabilitative treatment. Assessment of quality was restricted to the principal questions "Which patients are seeking treatment in the individual hospitals?" and "Which degree of improvement of the disease can be observed after rehabilitation?" The purpose of medical care is maintenance or improvement of health status for which outcome measures are of greater intrinsic interest [9], especially with respect to patients' perspective. As a consequence we focused in this study on the dimension of outcome quality although aspects of structure and process quality are essential components of a comprehensive quality profiling [10,11].

The objective of the study was to develop a proposal for methodology of outcome quality profiling, and to demonstrate a suitable technique for comparing quality of care of different providers. Potential benefit and limitations of such an approach are to be discussed by means of a case study on three real life examples in rehabilitative care.

## Methods

### Design

The project was designed as a prospective multi-center observational study with three repeated measurements per patient (at admission to the hospital, at discharge from the hospital and 6 months after discharge).

### Patients

Allocation of the patients to the hospitals followed the normal course of any individual provider. Eligible patients had to meet predefined inclusion/exclusion criteria focusing on diagnosis (migraine, tension-type headache or drug induced headache), disease duration (≥ 5 years) and degree of severity (≥ 5 days with headache per month). Diagnostic was supported by a standardized patient questionnaire (Kiel Headache Questionnaire [12]) built on the criteria of the International Headache Society. To avoid any in-house selection bias patients were to be enrolled consecutively during the period of recruitment (spring 2001 to autumn 2002) until targeted sample size was reached.

### Interventions

All patients underwent the in-patient treatment program usually applied in each hospital and described by already existing in-house guidelines. Participation in the study did not affect the choice of any therapeutic procedure. The respective basic treatment concepts of the three hospitals are characterized as follows:

• 1^st ^Hospital: specialized in treatment of headache with focus on withdrawal of pain killers. Holistic treatment concept following the recommendations of international guidelines (migraine prophylaxis and treatment of acute attacks), and including special diets, physiotherapy (hydrotherapy and massage), neural therapy, homeopathy, psychotherapy with relaxation techniques in compliance with individual indication.

• 2^nd ^Hospital: wide range of indications, about 12% of all patients treated suffer from chronic headache. For chronic headache comprehensive treatment concept including drug therapy and physical therapy (hydrotherapy, massage) as well as complementary methods like food therapy (fasting), acupuncture, cupping, neural therapy and supportive psycho-social group therapy (relaxation techniques and training in healthy life-style).

• 3^rd ^Hospital: about 20% of all patients suffer from chronic headache as the primary reason for the hospital stay, treatment concept based on traditional Chinese medicine. Patients with chronic headache receive 3 sessions of acupuncture and 2 sessions of tuina-massage per week on average, daily individualized Chinese drug therapy according to TCM syndrome diagnosis, wholesome nutrition, and qi-gong exercises twice a day.

All three hospitals provide treatment that generally has duration of 4 weeks. In this paper, the hospitals were arbitrarily designated A, B and C.

### Outcome quality profile and data collection

According to the holistic concept of CAM methods, outcome quality should be represented by a broad variety of indicators. Outcome measures were self-assessments by patients, and are summarized in Table 1.

**Table 1:** Overview of measures and times of administration

Variable	4 weeks before admission	Admission	Discharge	6 months after discharge
Pain intensity^1^		X	X	X
Pain disability^2^		X		X
Days with headache/with analgesics^3^	X			X
Affective pain^4^		X	X	X
Depression^5^		X	X	X
Physical and Mental Health^6^		X		X
Sense of coherence^7^		X		X
Satisfaction with health^8^		X	X	X
Health behavior^9^		X		X
Global assessment of treatment success^10^			X	X
Satisfaction with hospital stay^11^			X	
General health status^12^		X		
Internal health locus of control^13^		X		
Trust in successful treatment^14^		X		
Kiel Headache Questionnaire^15^	X			
Headache Anamnesis and diagnose^16^		X		

^1 ^Numeric rating scale from 0 = no pain to 10 = maximum pain (modified from the German Society for the Study of Pain survey [23])

^2 ^Sum score from German version of the Pain Disability Index PDI [24]

^3 ^Number of days during 4 weeks reported in headache diaries (administered once daily); headache day means day with pain intensity > 0

^4 ^Sum score (T-standard-value) "Pain affective" from the Pain Perception scale SES [25] for assessing emotional aspects of pain

^5 ^Sum score (T-standard-value) of the depression scale ADS [26], the German version of the Center for Epidemiological Studies Depression Scale

^6 ^Sum scores (T-standard-values) from the German version of the SF-36 [27] to assess health related quality of life

^7 ^Total score from a short form of the "Sense of Coherence Scale" SOC [28]

^8 ^Single item 'how satisfied are you with your health?' (5 categories from 1 = unsatisfied to 5 = very satisfied)

^9 ^4 sum scores for the domains 'healthy food' (frequency of consumption of different food, like fresh fruit), 'physical activity' (frequency of different activities, like walking, biking), 'coping with stress' (use of relaxation techniques), and 'self-help' (frequency of self-applications like cold water or wet packs), built from an ad-hoc 46-item questionnaire on health related behavior

^10 ^Single item with 5 categories (from 1 = very good to 5 = poor)

^11 ^7 sub scores based on factor analysis of an unpublished 35-item questionnaire to assess satisfaction of in- patients

^12 ^Item 1 from the SF-36, assessing the current subjective status of health (5 categories from 1 = bad to 5 = excellent)

^13 ^Single item to capture the amount of self control of the course of the disease (5 categories from 0 = not at all to 4 = very strong)

^14 ^Single item to estimate belief in a successful therapy (5 categories from 0 = not at all to 4 = very strong)

^15 ^Patient questionnaire with 37 items [12]

^16 ^Documented by physician using an electronic software system (Medical Monitoring [13])

Besides the assessment of treatment success and patient satisfaction with hospital stay outcomes were defined as differences between measurements at baseline and discharge from hospital and 6 months later, respectively.

In each hospital patients with a planned admission were screened for suffering from headache on the basis of their registration forms. If so, about 4 weeks prior to admission, patients were sent the Kiel Headache Questionnaire as well as a headache diary; both of which were to be completed before arrival at the hospital. In case of inclusion, patients filled out questionnaires assessing baseline status within the first 2 days of their hospital stay. At discharge, only those questionnaires which do not refer to everyday life conditions were administered a second time (see Table 1). Additionally, a scale for assessing the patient's satisfaction with hospital stay was attached. Six months after being discharged, patients were sent a third questionnaire and another headache diary by mail. The completed questionnaires could be returned to the hospital at no cost to the patient. In the case of missing follow-up data, patients were reminded by mail or phone (≤ 2 reminders altogether). Medical data (data on headache-related history and diagnosis) were documented by an electronic software system (Medical Monitoring [13]) that was available in all three hospitals. Recruitment started in spring 2001, and collection of follow up data was terminated in summer 2003.

All patient data were forwarded anonymously to the coordinating study center for statistical analysis. An ethical review board was not involved because of the strictly routine character of the study as part of a quality assurance program. All legal obligations for the protection of personal data were met, and patients gave written informed consent.

### Statistics

To visualize different outcomes in terms of a profile, observed values were transformed to standard z scores (with mean = 0 and standard deviation = 1) using the overall means and standard deviations for the total patient group before statistical testing. Effect sizes were used to translate the before-after differences into a standard unit by dividing the mean difference by the standard deviation of the difference [14].

Statistical analysis was explorative; hence results of statistical tests are not interpreted as hypothesis testing. It focused on analyzing differences between the three hospitals (first main factor) with the difference scores (admission/discharge and admission/6 months after, respectively) of the outcome measures as dependent variables. To test for differences between diagnostic subgroups (migraine alone versus tension-type headache and combination of both, respectively) this factor was also represented within the general linear model (second main factor). Basic descriptive variables, baseline values and outcome measures were tested for differences with regard to the two main factors "Hospital" and "Diagnostic Subgroup" and the interaction effect. To adjust for differences in baseline characteristics amongst patient groups, variables with statistically significant (p < .05) findings for the factor "Hospital" (age, education, internal health locus of control, trust in successful treatment, previous treatment with complementary/alternative methods, baseline values for pain intensity, for days with headache and for the corresponding outcome, if appropriate, plus general health) were added as covariates to the statistical model (analysis of covariance). In case of a statistically meaningful "Hospital" effect (p < 0.05, 2-sided) concerning the outcomes, subsequent pair-wise comparisons with Bonferroni correction to adjust for error probability were provided.

The estimated impact of data loss for the follow-up query was exemplified by the outcome measure 'number of days with headache' (responder: at least 50% reduction with regard to baseline). Patients without respective data were assigned as non-responders.

According to the explorative character of the analysis, sample size estimation was based on pragmatic aspects like recruitment, expected study duration and expenditure. Thus, a sample size of 125 patients per study center was deemed adequate to reveal fairly reliable estimates for the described differences.

## Results

### Data overview

A total of 567 patients were screened in the participating hospitals for enrollment in the study. In hospital A, 126 patients were eligible for the study, hospital B had 151 eligible patients, and hospital C had 103 (Table 2). All patients with available data from the questionnaires both at admission and at discharge and a completed baseline headache diary were included in the statistical analysis. 306 patients met these conditions. The proportion of patients with available follow-up data (six months after discharge) differed amongst the three hospitals and ranged from 55.0% in hospital B and 59.6% in hospital A to 83.7% in hospital C (p < .05).

**Table 2:** Overview of data structure

	Number of patients		
	**Hospital A**	**Hospital B**	**Hospital C**
Screened for study	246	192	129
			
Meeting the inclusion/exclusion criteria	126	151	103
Missing data:			
- Patient questionnaire missing at admission*	3	20	0
- Patient questionnaire missing at discharge*	14	14	5
- Baseline diary missing*	12	11	1
			
For main statistical analysis**	**99**	**109**	**98**
			
With 6-month follow-up data including headache diary	59	60	82

* multiple specifications possible

** patient questionnaires at admission, at discharge and baseline headache diary mandatory

### Patients

Nearly all patients (92.8%) were assigned the diagnosis "migraine" and 45.4% had "tension-type headache" (not significantly different amongst the hospitals). The proportions of patients with drug-induced headache were 2% in hospital C and 23% each in hospitals A and B (p < .01). Since more than one diagnosis was possible for diagnostic classification, the rates for the single diagnoses do not add up to 100 percent. As there were clear differences for the distribution of diagnoses amongst the hospitals (p < .01), we decided to split the patients into two subgroups for the statistical analyses: one group included patients with just migraine (54.6%) and the other included patients with tension-type headache or a combination of both (45.4%).

The mean age of all patients was 48.4 (sd 11.1) years, the majority of patients were women (88.6%). Amongst the patients of the hospitals we observed statistically significant differences in age, education level, use of drugs for acute headache, previous experience with complementary and alternative methods (CAM), level of internal health locus of control, and in the degree of trust in a successful treatment (Table 3). Furthermore, the diagnostic subgroups differed clearly with regard to subjective general health status and to the way patients treat acute headache. Significant interaction effects indicate that, for example, health status amongst patients of both headache groups was differently distributed within the three hospitals. Further examples were previous CAM experience and internal health locus of control.

**Table 3:** Patients' profile for socio-demographic and medical history data; split for hospital and diagnostic subgroup (TTH = tension-type headache; m = arithmetic mean, sd = standard deviation); - not significant, * p < 0.05, ** p < 0.01

		Providing hospital			Diagnostic headache subgroup			Significance testing		
		Hosp. A	Hosp. B	Hosp. C	Migraine alone	TTH or combined with migr.	Total	Between	Between	Hosp.X
	N =	99	109	98	167	139	306	Hosp.	Subgr	Subgr
Age	Years; m (sd)	51.8 (9.6)	46.3 (11.1)	47.3 (12.0)	48.6 (10.6)	48.2 (11.8)	48.4 (11.1)	**	-	-
Gender	Female %	88.9	90.8	85.7	90.4	86.3	88.6	-	-	-
Education	>12 school years %	24.2	34.9	18.4	25.1	27.3	26.1	*	-	-
Occupational status	Employed %	63.6	67.9	65.3	66.5	64.7	65.7	-	-	-

Headache since	Up to 5 yrs %	3.0	5.6	3.1	3.0	5.1	3.9			
	6 to 10 yrs %	13.1	10.2	14.3	10.8	14.5	12.5			
								-	-	-
	11 to 20 yrs %	17.2	22.2	22.4	22.8	18.1	20.7			
	> 20 yrs %	66.7	62.0	60.2	63.5	62.3	63.0			

Prophylactic treatment	Drug treatment %	34.1	38.5	26.6	33.5	32.8	33.2	-	-	-
	Non-drug %	73.9	67.0	70.8	73.2	66.9	70.4	-	-	-
Acute headache	Drug treatment %	97.8	99.1	89.2	96.8	94.0	95.5	**	-	-
	Non-drug %	47.2	49.5	51.2	44.4	54.8	49.3	-	-	-
Previous treatment	with CAM§methods %	59.1	79.0	83.2	77.6	69.7	74.2	**	-	*

General health status	m (sd)	2.50 (0.88)	2.61 (0.95)	2.48 (1.13)	2.64 (1.01)	2.40 (0.94)	2.53 (0.99)	-	*	*
Internal Health locus of control	m (sd)	1.87 (0.84)	1.83 (0.93)	1.48 (0.98)	1.70 (0.85)	1.76 (1.02)	1.73 (0.93)	**	-	*
Trust in successful treatment	m (sd)	3.01 (0.76)	3.31 (0.77)	3.44 (0.61)	3.32 (0.69)	3.18 (0.78)	3.26 (0.74)	**	-	-

§ Complementary and Alternative Medicine

The observed significant differences amongst the hospitals for several variables caused us to consider them, in addition to the corresponding baseline values (see below), as potential covariates for testing outcomes.

### Baseline status

Tests for baseline differences both amongst the hospitals and between the diagnostic subgroups revealed a large number of statistically significant differences for the patients from the three hospitals concerning pain intensity, number of days with headache, depression, sense of coherence, satisfaction with own health and food habits (Table 4). Patients' profiles from hospital C could be characterized as showing the highest average pain intensity, most headache days per month, highest depression, poorest sense of coherence, lowest health related satisfaction as well as poorest food habits. Patients in the subgroup "migraine" had the worst intensity of pain and suffered from a higher degree of disability although they experienced fewer days with headache, were less depressed and showed a better mental health rating.

**Table 4:** Comparison of baseline values at admission to the hospital, split for hospital and diagnostic subgroup (TTH = Tension-type headache; m = arithmetic mean, sd = standard deviation); - not significant, * p < 0.05, ** p < 0.01

		Providing hospital			Diagnostic headache subgroup			Significance testing		
		Hosp. A	Hosp. B	Hosp. C	Migraine alone	TTH or combined with migr.	Total	Between	Between	Hosp. X
	n =	99	109	98	167	139	306	Hosp.	Subgr	Subgr
Pain intensity	m (sd)	5.8 (1.5)	5.7 (1.7)	6.5 (1.8)	6.2 (1.8)	5.7 (1.5)	6.0 (1.7)	**	*	-
Pain disability Index	m (sd)	35.3 (15.7)	36.6 (15.6)	38.9 (15.3)	39.9 (16.4)	33.4 (13.6)	37.0 (15.5)	-	**	-
Days with headache	Per 4 weeks; m (sd)	12.0 (5.5)	12.8 (6.2)	14.8 (7.0)	11.4 (5.5)	15.3 (6.7)	13.2 (6.4)	**	**	*
Days with analgesics	Per 4 weeks; m (sd)	7.8 (4.8)	9.5 (6.3)	9.0 (6.4)	8.7 (5.7)	8.9 (6.2)	8.8 (5.9)	-	-	-

Pain affective	T-values; m (sd)	58.6 (8.5)	56.9 (7.9)	57.5 (7.6)	58.8 (8.6)	57.7 (7.7)	58.3 (8.2)	-	-	-
Physical Health#	T-values; m (sd)	38.7 (8.0)	38.4 (7.7)	36.8 (8.9)	38.0 (8.2)	38.0 (8.2)	38.0 (8.2)	-	-	-
Mental Health#	T-values; m (sd)	42.0 (11.5)	43.4 (11.4)	42.0 (10.8)	43.7 (10.9)	41.1 (11.5)	42.5 (11.2)	-	*	-
Depression	T-values; m (sd)	53.8 (8.5)	51.9 (9.4)	54.7 (9.7)	52.1 (9.3)	55.1 (9.0)	53.4 (9.2)	*	**	-
Sense of coherence#	m (sd)	64.2 (11.8)	65.7 (11.9)	61.2 (13.3)	64.8 (11.9)	62.6 (13.0)	63.8 (12.4)	*	-	-
Satisfaction with health#	m (sd)	2.1 (0.9)	2.1 (1.0)	1.8 (0.9)	2.0 (0.9)	2.0 (0.9)	2.0 (0.9)	*	-	*

Healthy food#	m (sd)	11.6 (2.8)	10.9 (2.6)	10.5 (2.4)	11.0 (2.5)	11.0 (2.8)	11.0 (2.6)	*	-	-
Physical activity#	m (sd)	5.9 (2.1)	5.6 (2.5)	5.4 (2.3)	5.7 (2.3)	5.6 (2.3)	5.6 (2.3)	-	-	-
Coping with stress#	m (sd)	3.4 (2.2)	3.2 (2.2)	3.1 (2.2)	3.2 (2.2)	3.3 (2.2)	3.2 (2.2)	-	-	-
Using of self-help#	m (sd)	0.9 (1.1)	0.7 (0.9)	0.8 (0.9)	0.9 (1.0)	0.7 (0.9)	0.8 (1.0)	-	-	-

# higher values indicate better status

### Treatment outcomes

**Figure 1:**
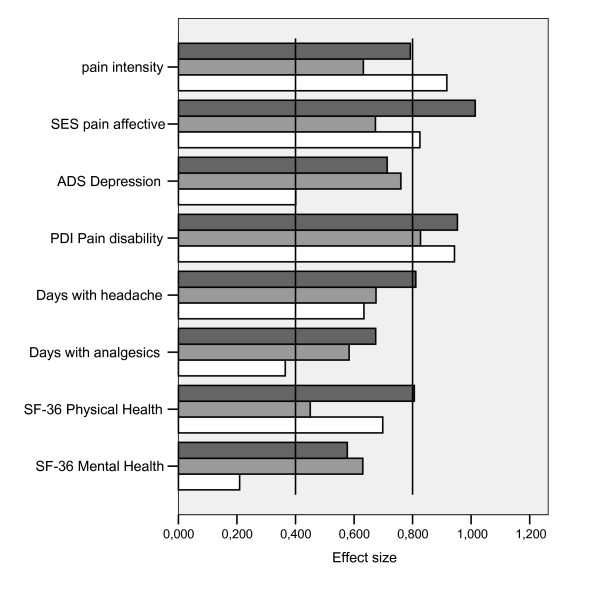
Effect sizes adjusted for covariates for differences between baseline and 6 month follow-up query; dark grey: Hospital A, grey: Hospital B, white: Hospital C (effect sizes below 0.4 indicate small effects, between 0.4 and 0.8 moderate effects and greater than 0.8 large effects).

At discharge from the hospital, patients generally benefited markedly from treatment with regard to all outcome measures for which data had been collected at this point. Six months later, most outcomes showed effect sizes that fell in the range of moderate effects (0.4 to 0.8), indicating lasting and clinically relevant improvements (Fig. 1). This finding was true for all three hospitals. The lowest effect sizes were associated with the indices of health behavior like physical activity or food habits.

### Outcome profiles

At discharge from the hospital, after controlling for the list of covariates, there was a statistically significant difference (p < .01) amongst the hospitals in patients' global assessment of treatment success (Table 5). Patients from hospital B rated the treatment more positively than those from hospital A and those from hospital C. More significant differences were found concerning the subscores for patients' satisfaction with the hospital stay. For this parameter, patients from hospital C were less satisfied in most of the single dimensions of satisfaction than patients from the other hospitals.

**Table 5:** Results for testing the effects for outcome measures with respect to the factors "Hospital", "Diagnostic subgroup" as well as to the interaction term adjusted for covariates (Analysis of Covariance); - not significant, + p < 0.10, * p < 0.05, ** p < 0.01

Outcome measures	Factor "Hospital"		Factor "diagnostic subgroup"		Interaction "Hosp. X Subgroup"	
	Discharge	6-m follow-up	Discharge	6-m follow-up	Discharge	6-m follow-up
Pain intensity#	-	-	*	-	-	-
Pain affective#	-	-	-	-	-	-
Depression#	-	-	+	-	-	-
Global assessment of success	** ^1,2^	-	*	-	-	-

Pain disability#		-		-		-
Physical health#		+		-		-
Mental health#		+		-		-
Days with headache#		-		+		-
Days with analgetics#		-		+		-
Sense of coherence#		-		-		-
Satisfaction with health#		-		-		-
Healthy food#		-		-		-
Physical activity#		-		-		-
Coping with stress#		* ^2^		-		-
Using self-help#		** ^1,3^		**		-

Patients satisfaction scores:						
Nurses care	-		-		-	
Physicians care	** ^1,2^		-		-	
Other therapies	** ^2,3^		-		-	
Health promoting	** ^2,3^		-		-	
Hospital's equipment	** ^1,2^		-		-	
Diverse aspects	*		-		-	
Information	-		-		-	
Satisfaction sum score	* ^3^		-		-	

#Differences from baseline

Posteriori comparisons (p < .05, Bonferroni adjusted) between hospitals: ^1^A-B ^2^B-C ^3^A-C

Six months after discharge from the hospital, statistically significant differences could only be found for two sub-indices of health behavior. Patients from hospital B showed greater improvement in coping with stress, and patients from hospital A were more successful in increasing their use of self-help. There was a trend for significant differences (p < .10) amongst the hospitals concerning the increase in physical and mental health, favoring patients from hospital A and B, respectively.

For the migraine subgroup, statistically significant differences could be found for a greater decrease of pain intensity as well as a better overall rating of treatment success at discharge from the hospital (Table 5). 6 months later, patients with tension-type headache (alone or in combination with migraine) showed a more pronounced increase in their use of self-help than patients with migraine alone. The overall comparison of the three hospital groups revealed a statistical trend to significant differences concerning the total scores from SF-36. Testing for interaction effects amongst the "Hospital" and the "Diagnostic subgroup" did not show a statistically significant result for any single outcome measure.

**Figure 2:**
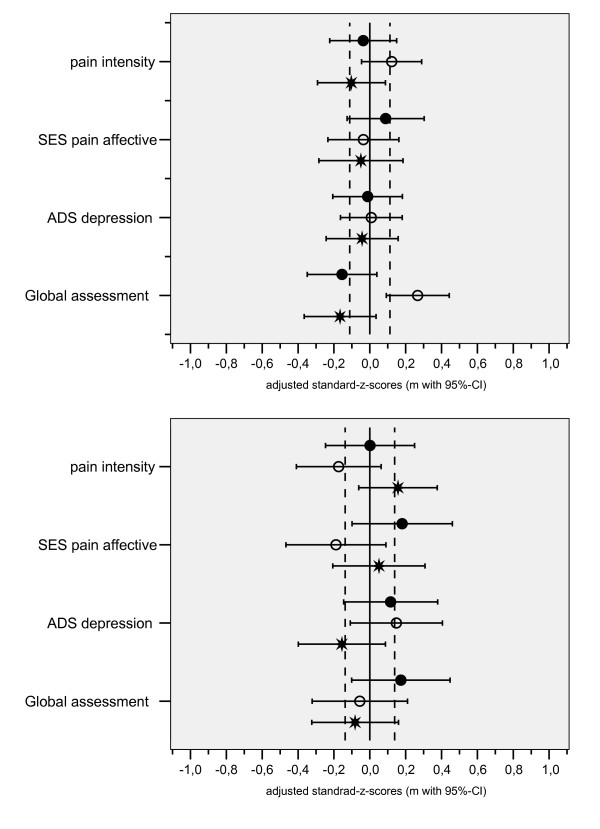
Profile of outcome quality consisting of four outcome measures at discharge from hospital (above) and 6 months after (below); the graph represents mean z-scores (with 95% confidence interval) adjusted for covariates, values right of the vertical zero line mean better results compared to the grand mean of all three hospitals (• hospital A, ° hospital B, * hospital C).

The graphical representation of outcome profiles was designed to expose differences between hospitals (Fig. 2). Here, the mean deviations of each hospital's patient group for several distinct outcomes are shown in relation to a reference range representing the grand mean (with 95% confidence interval) of all pooled patients. The given values are standardized z-scores with their respective 95% confidence intervals adjusted for covariates.

Figure 2 shows, with one exception, only slight differences in outcomes amongst the patient groups at discharge from the hospital. Six months later, the same outcomes led to a more heterogeneous pattern but failed to fulfill the criteria for statistical significance.

Calculated on the basis of patients with available data, the proportion of patients that responded to treatment 6 months after discharge (at least 50% reduction in headache days) was 40.7% from hospital A, 35.0% from B and 41.5% for C. Assuming that patients who did not participate in the follow-up query were non-responders, the modified percentages were 24.2% for patients from hospital A, 19.3% for B and 34.7% for hospital C.

**Figure 3:**
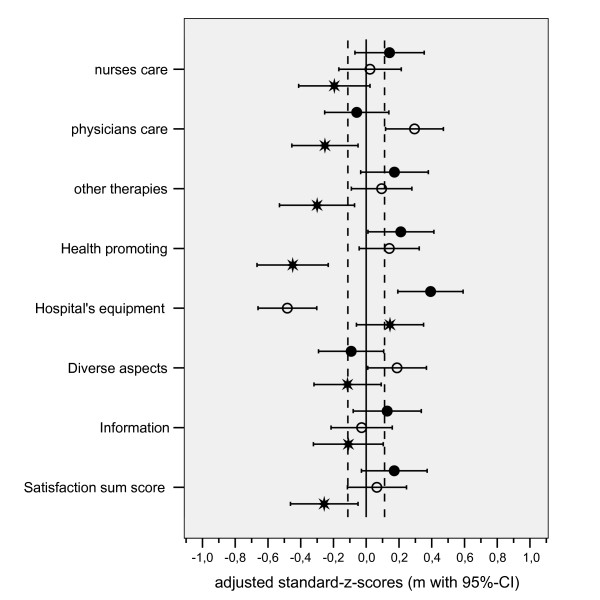
Profile of different components of patients' satisfaction at discharge from the hospital; the graph represents mean z-scores (with 95% confidence interval) adjusted for covariates, values right of the vertical zero line mean better results compared to the grand mean of all three hospitals (• hospital A, ° hospital B, * hospital C).

The final part of the outcome profile examines patients' satisfaction with the hospital stay. The greatest differences amongst the hospitals were seen for this aspect of the profile (Fig. 3). While patients from hospital C showed poorer adjusted satisfaction scores for most dimensions, satisfaction with the hospital's equipment was better than the average of all patients. Conversely, patients from hospital B had the lowest scores for this parameter.

## Discussion

Illustrated by in-patients with chronic headache, our comparative case study provided quality profiles with regard to patients' characteristics and treatment outcomes for three hospitals providing non-conventional rehabilitative care. This approach, however, is only one part of a more comprehensive provider profiling which also includes aspects of infrastructure, intervention and activities in quality improvement. It should be pointed out that our results reflect the routine situation in rehabilitative care of three defined providers selected for this case study. The 'natural' way of patient selection and entanglement of treatment concept with the provider's way of putting it into practice are a matter of fact in daily medical care.

Reliability and validity of the given results are the crucial factors. As to the first point, we regard the sample size as adequate to allow reliable estimates of treatment success with reasonable confidence intervals. Nevertheless the study might be underpowered, and it should be pointed out that all findings concerning statistically meaningful differences are related to the given sample size.

The samples reflected the 'average' headache patient of the hospitals with a confirmed primary indication for rehabilitation. For the sickness funds 'migraine' is usually accepted as indication which explains that nearly all patients of the sample being assigned this diagnosis. The high decline in the number of eligible patients after applying the inclusion criteria was due to the 4-week period prior to admission to the hospital when recruitment was only possible by means of unstructured paper documents. The mandatory baseline phase also resulted in the patients not being included in the study in a strictly consecutive manner. Problems in scheduling the hospital stay prevented several patients from participating. There was no bias for selection within each hospital as inclusion was not suspected to be dependent on the expected success of the therapy. A more serious problem arose from attrition during the follow-up period. While the rate of patients with available query data was barely sufficient at two hospitals (55 to 60%), patients from the other hospital showed a distinctly better rate (approx. 84%). This hospital had already implemented a routine follow-up query since several years where patients proved to be in closer relationship with the hospital. Whenever willingness to participate and the success of the intervention are suspected to be not independent, the possible impact of different participation rates must be taken into account. Declaring non-participants as treatment failures thus led to quite different results. Patients with available follow-up data were more likely to be female and to show lower education level. However, no other statistically significant differences in baseline measures could be detected between patients with and without follow-up data, respectively. Anyway, to avoid uncertainty by missing data more efforts should be made in future to minimize this weakness.

First analyses showed that patients from the three hospitals were different in several respects, thus violating the precondition for direct outcome comparisons. By performing analyses of covariance we chose a linear regression analysis based model in order to adjust for these differences. We did not calculate propensity scores [15] because both methods resulted in similar conclusions [16,17]. Instead, we considered the definition of the relevant adjustment factors and the choice of the indicators of outcome quality to be more important. Random assignment of a sufficiently large sample of patients to the three providers would have minimized the likelihood for structural inequalities. However, such an approach failed because of the common administrative rules of the rehabilitation system. Moreover, this kind of 'experimental' comparison would be invalid with regard to everyday situation [18], as sickness funds usually try to honor patients' preference for a certain hospital.

For treatment of chronic headache, there is a consensus with respect to several important outcome measures, like reduction in pain intensity, decrease in days with headache, and decrease in pain-induced functional disabilities. Rehabilitative interventions include a broader range of factors affecting outcomes of care [19] and there is demand for further outcome dimensions according to different intervention goals. This approach is in line with the holistic concept of CAM methods which also takes into account health-related psychosocial factors. That is why we expanded the outcome profile to include other factors, like quality of life, life satisfaction, sense of coherence and selected items of health behavior. Though for many clearly defined medical interventions there is an accepted standard for outcome quality, this is not true for the rehabilitation of headache sufferers. Our quality profile may not represent all of the potential quality indicators, but it should be noted that our proposal included several well accepted assessment instruments and a standardized computer-based medical documentation system.

Statistical analysis concentrated on the comparison of the results from the three hospital groups. Based on clinically relevant improvements in headache-associated outcomes which could be observed similarly in all patient cohorts from the three hospitals, differences amongst hospitals were rather minor. After adjustment for covariates, the only significant result was a better global assessment of the therapeutic effect at discharge by the patients from hospital B. 6 months later; this effect was no longer present. On the other hand, follow-up data showed that the patients' capacity in coping with stress improved the most in patients from hospital B, and the usage of self-help in everyday life changed in favor of patients from hospital A. Both findings reflect crucial points of the treatment programs practiced in the corresponding hospitals. More information on what happened to the patients after discharge from the hospital would be necessary for a better understanding of the different findings on short and middle term effects. The diagnostic subgroup was an important factor for the description of the patients and baseline comparisons, but did not seem to play a role in treatment results. Furthermore, it did not interact with the factor "hospital", which means that both headache groups benefited similarly in each hospital. The patients' satisfaction at discharge from the hospital turned out to be the most sensitive outcome. Hospitals were characterized by clearly distinguishable profiles that indicated that patients felt more satisfied with certain aspects in one hospital and with other aspects in the other hospitals.

In the end, our case study offers more transparency regarding the type of patients that came to the different hospitals for treatment and the results that could be achieved. Quality profiles can be roughly screened for relevant differences a) between two hospitals whenever their confidence intervals do not overlap, and b) with regard to a reference (perfectly a gold-standard, if available) whenever the confidence interval of one hospital lies completely outside the reference range (in our case pooled patients from all hospitals). Using standardized scores for the different outcome measures, results are directly comparable and independent of different units and variances. However, it should be noted that this is only valid in the context of this case study. Repetitions of the study in these hospitals as well as in newly investigated providers would mean another point of reference for standardization. More rigorous data would be necessary to claim for a reliable and stable standard of evaluation.

Comparisons with the outcomes of patients from conventional headache units would definitely enrich our analyses, yet an in-patient treatment is usually not available in such facilities. For ambulant patients recent large trials on acupuncture in Germany revealed similar effects in migraineurs [20,21] but comparison with our patients appears to be questionable.

## Conclusion

Our proposal should contribute to the discussion of the right way to comply with the legal requirements of comparative quality assurance. Chances and limitations of such analyses demonstrate that comparing the quality of medical care by league tables [22] poses a challenge both from a methodological and a clinical perspective. Although there was not a clear winner or loser in the comparison of the three providers treating in-patients with chronic headache, the complex pattern of such outcome profiles should aid decision-makers about which hospital might have priority. Thereby preferences may be based on different subjectively weighted aspects of quality. Whatever the background and the motivation for questions addressed to the providers may be, a transparent database like this one should make finding answers easier.

## Competing interests

The author(s) declare that they have no competing interests.

## Authors' contributions

DM initiated the study, led the development of the protocol, and contributed to interpretation of the results. AW, SH and RB were responsible for recruitment of patients and for daily management of the study, WW performed the statistical analysis and drafted the paper. All authors read and approved the final manuscript.

## Pre-publication history

The pre-publication history for this paper can be accessed here:


